# Electromicrobiological concentration cells are an overlooked potential energy conservation mechanism for subsurface microorganisms

**DOI:** 10.3389/fmicb.2024.1407868

**Published:** 2024-08-21

**Authors:** Ian P. G. Marshall

**Affiliations:** Center for Electromicrobiology, Department of Biology, Aarhus University, Aarhus, Denmark

**Keywords:** concentration cells, cable bacteria, microbial ecology, metabolism, electromicrobiology

## Abstract

Thermodynamics has predicted many different kinds of microbial metabolism by determining which pairs of electron acceptors and donors will react to produce an exergonic reaction (a negative net change in Gibbs free energy). In energy-limited environments, such as the deep subsurface, such an approach can reveal the potential for unexpected or counter-intuitive energy sources for microbial metabolism. Up until recently, these thermodynamic calculations have been carried out with the assumption that chemical species appearing on the reactant and product side of a reaction formula have a constant concentration, and thus do not count towards net concentration changes and the overall direction of the reaction. This assumption is reasonable considering microorganisms are too small (~1 μm) for any significant differences in concentration to overcome diffusion. However, recent discoveries have demonstrated that the reductive and oxidative halves of reactions can be separated by much larger distances, from millimetres to centimetres via conductive filamentous bacteria, mineral conductivity, and biofilm conductivity. This means that the concentrations of reactants and products can indeed be different, and that concentration differences can contribute to the net negative change in Gibbs free energy. It even means that the same redox reaction, simultaneously running in forward and reverse, can drive energy conservation, in an ElectroMicrobiological Concentration Cell (EMCC). This paper presents a model to investigate this phenomenon and predict under which circumstances such concentration-driven metabolism might take place. The specific cases of oxygen concentration cells, sulfide concentration cells, and hydrogen concentration cells are examined in more detail.

## 1 Introduction

Microbial communities inhabit spatially heterogeneous and complex environments that resist simple understanding. While most of our understanding of microbial physiology is the result of more than a century of cultivating and isolating pure cultures of microorganisms, this approach has proven inadequate for understanding all microbial diversity, with 22–87% of archaeal and bacterial genera remaining uncultivated depending on the environment (Lloyd et al., 2018). Spatially heterogeneous environments such as sediment, the terrestrial subsurface, and soil contain the highest fractions of uncultivated taxa (Lloyd et al., 2018). Such spatially heterogeneous environments contain concentration gradients of microbial substrates, such as O_2_, sulfide, and H_2_. It has recently been shown that certain microorganisms can link metabolic reactions taking place across these gradients via long-distance electron transport between microbial cells, thus allowing them to better exploit optimal substrate concentrations than a single cell. One example of this is the cable bacteria *Electronema* and *Electrothrix* (Trojan et al., 2016; Plum-Jensen et al., 2024), which were discovered through careful measurement of chemical microprofiles in aquatic sediment (Pfeffer et al., 2012). Cable bacteria are filamentous bacteria that couple the oxidation of sulfide in deep, anoxic sediment to the reduction of oxygen in shallow sediment. They conduct electricity over several centimetres through as-yet uncharacterized conductive structures in their periplasm. This discovery changed our conception of what living organisms are capable of – we previously thought that oxidative and reductive halves of an organism’s metabolism had to exist in the same cell, cable bacteria demonstrated that it is possible to oxidize an electron donor in one cell and use that electron to reduce an electron acceptor in another cell, possibly centimetres away, by transporting that electron through a conductive biological wire much faster than diffusion could transport an electron carrier molecule. Similarly, it has been shown that microorganisms can transfer electrons centimetre distances via networks of dissolved organic matter and organo-mineral associations (Bai et al., 2023), or across millimetre distances in biofilms (Li et al., 2016).

There is also increasing evidence that some anaerobic microorganisms can reverse their metabolism depending on the surrounding chemical environment. For example, acetogens have been shown to oxidize acetate in a H_2_-consuming co-culture (Hattori et al., 2005). Obviously, a single-celled microorganism cannot conserve energy through the simultaneous oxidation and reduction of the same compound – this reaction would not be exergonic. However, if we combine this concept with the long-distance transport of electrons one can imagine two distant, yet electrically connected, bacterial cells in different chemical environments – one environment with a higher concentration of electron donor favouring the forward reaction and the other with a higher concentration of the oxidized form of the substrate favouring the reverse reaction. The metabolism of these microorganisms would be powered by an electrochemical concentration cell.

An electrochemical concentration cell is an electrochemical system where a single redox reaction, run both in forward and reverse directions, can result in an electrical current between two different concentration regimes (Foulkes, 2012). Such concentration cells are well known in some fields, such as the contribution of oxygen concentration cells to metal corrosion (Iverson, 1987). Traditionally, concentration cells have not been seen as relevant for individual microorganisms, as a ~1 μm cell is much too small to span multiple concentration regimes. However, the discovery of long-distance electron transport in heterogeneous subsurface environments by cable bacteria, conductive minerals, and biofilms makes this topic newly relevant. Concentration differences could drive microbial metabolism in the subsurface in a way that has not been previously recognized, in what could be called an ElectroMicrobiological Concentration Cell (EMCC). The energy yields would be small, and turnover rates may be small compared to other processes driving the concentration differences, but the energy-starved subsurface has previously shown to be homes for such low-energy-yield metabolisms, like anaerobic methane oxidation (Knab et al., 2008) or acetogenesis (Lever, 2012), and low metabolic rates are also common in subsurface environments (Jørgensen and Marshall, 2016). As long as steep concentration gradients exist, for example at geochemical transition zones or boundaries between sediments or rocks of different chemical compositions, then EMCCs are thermodynamically possible. The minimum energy requirement to support oxidative phosphorylation is thought to be as low as −10 kJ/mol (Hoehler et al., 2001). At least one microbial cell involved in the reaction would have to conserve energy at any given time, and if multiple cells conserved energy simultaneously this would multiply energy needs. There may also be situations where such concentration cells do not support microbial energy conservation, but their activity still may impact geochemical element cycling.

The goal of this paper is to develop a theoretical framework for understanding microbial metabolism through concentration cells. I will present three concentration-cell scenarios based on H_2_O/O_2_, SO_4_^2−^/S^2−^, and H^+^/H_2_.

## 2 Model

We will assume that this reaction takes place in two distant yet electrically connected cells, one in a more reducing environment and one in a more oxidizing environment.

The reaction at the cell in the reduced compartment is the following:


$${\mathrm{XH}}_{n}\to X+nH^{+}+ne^{-}$$

Where X is the oxidized form of the substrate, XH_*n*_ is the reduced form of the substrate, n is the number of electrons transferred, and H^+^ indicates a proton. The electrons from this reaction are transferred onto a conductor, leading to one or more cells in the oxidized compartment where the reverse reaction takes place:


$$ne^{-}+nH^{+}+X\to {\mathrm{XH}}_{n}$$

These two reactions can be combined to show the sum of both reactions across the reducing and oxidizing environments in a galvanic cell. Subscripts “*ox*” and “*red*” will be used to keep track of where each chemical species is produced or consumed (not whether it is reduced or oxidized). Electrons do not appear in this equation, as electrons produced on the left-hand side are the consumed on the right hand side.


$${\mathrm{XH}}_{n,red}+{\mathrm{nH}}_{ox}^{+}+X_{ox}\to {\mathrm{XH}}_{n,ox}+nH_{red}^{+}+X_{red}$$

The next step is to determine whether this reaction can proceed exergonically, and what the Gibbs free energy is. While there is no change in Gibbs free energy from the substrates to the products, as the total Gibbs free energy of formation remains unchanged with identical chemical species as substrates and products, the change in energy is purely driven by concentration differences between the reducing and oxidizing environment:


$$\Delta G=RT\ln\left(\frac{\left[{\mathrm{XH}}_{n,ox}\right]{\left[H_{red}^{+}\right]}^{n}\left[X_{red}\right]}{\left[{\mathrm{XH}}_{n,red}\right]{\left[H_{ox}^{+}\right]}^{n}\left[X_{ox}\right]}\right)$$

Where ΔG is the change in Gibbs free energy, *R* is the gas constant, *T* is temperature in kelvin, and *ln* indicates the natural logarithm. A negative ΔG will result in an exergonic reaction, which means, generally speaking, that as long as the numerator is less than the denominator in the above equation the reaction can proceed. For simplicity, all models in this paper will only alter the value of a single chemical species between the reducing and oxidizing environment. These concentration differences (and similarities) must be maintained by other biological, chemical, or physical processes for the reaction to proceed. Differences in pH between the two environments can be critical for whether the reaction can proceed.

There is one final element to add to this model, and that is energy loss between the reducing and oxidizing environment as a result of resistance. We assume a linear drop in potential with distance:


$$\Delta G=RT\ln\left(\frac{\left[{\mathrm{XH}}_{n,ox}\right]{\left[H_{red}^{+}\right]}^{n}\left[X_{red}\right]}{\left[{\mathrm{XH}}_{n,red}\right]{\left[H_{ox}^{+}\right]}^{n}\left[X_{ox}\right]}\right)+nFED$$

Where *n* is the number of electrons transferred per reaction, *F* is Faraday’s constant, *E* is the expected voltage drop per unit distance, and *D* is the distance. *E* has been estimated for cable bacteria to be 12.3–14.6 ± 3.8–4.1 mV mm^−1^ (Bjerg et al., 2018), so I use a value of 13 mV mm^−1^ in this paper. Other conductors, such as biofilms or organo-mineral associations, may have different values.

Another way of expressing this model is as a function of potentials for the reactions where XH is oxidized and where X is reduced:


$$\Delta E=E_{red}+E_{ox}-E_{loss}$$

Each chemical species contributes to the overall potential change, along with the loss of some potential in along the conductor. Each of these has a standard potential modified by the local concentrations of all products and substrates. This is where XH is oxidized:


$$E_{ox}=-E^{0}-\left(\frac{RT}{nF}\right)\ln\left(\frac{{\left[H_{red}^{+}\right]}^{n}\left[X_{red}\right]}{\left[{\mathrm{XH}}_{n,red}\right]}\right)$$

And where X is reduced:


$$E_{red}=E^{0}-\left(\frac{RT}{nF}\right)\ln\left(\frac{\left[{\mathrm{XH}}_{n,ox}\right]}{{\left[H_{ox}^{+}\right]}^{n}\left[X_{ox}\right]}\right)$$

Let us take an example of an O_2_/H_2_O EMCC, where the O_2_ concentration at the oxidized end is 300 μM, at the reduced end is 1 nM, and where pH is a constant 7. E^0^ for O_2_ reduction to H_2_O is 818 mV for O_2_ in an aqueous solution (Thauer et al., 1977). The pH is 7 at both ends, and water is not included in the reaction quotient as this is already taken into account by the standard potential for an aqueous solution. For the oxidation reaction, the standard potential needs to be made negative to show its reversal relative to the reduction reaction. There is 2 mm of conductor between the two points.


$$E_{ox}=-0.818-\left(\frac{\left(8.314\right)\left(293\right)}{\left(4\right)\left(96485.3\right)}\right)\ln\left(\frac{{\left(10^{-7}\right)}^{4}\left(10^{-9}\right)}{1}\right)$$

$$E_{ox}=-1.276\;V$$

$$E_{red}=0.818-\left(\frac{\left(8.314\right)\left(293\right)}{\left(4\right)\left(96485.3\right)}\right)\ln\left(\frac{1}{{\left(10^{-7}\right)}^{4}\left(0.0003\right)}\right)$$

$$E_{red}=1.356\;V$$

$$E_{loss}=0.013*2\mathrm{mm}$$

$$E_{loss}=0.026V$$

ΔE=1.356V−1.276V−0.026V

ΔE=0.054V

ΔG=−nFΔE

ΔG=(−4)(96485.3)(0.054)

ΔG=−20.8kJ/mol

To model these electromicrobiological concentration cells (EMCCs), I assume that one reaction takes place in a single point in one-dimensional space and then calculate ΔG for cells electrically connected to this point. The idea that one half of the reaction takes place in a very small area and the other half over a larger area is consistent with our current understanding of cable bacteria, where oxygen reduction is restricted to <10% of the filament (Scilipoti et al., 2021).

Real-world pH and O_2_ microprofile data was obtained from the Pangaea database from studies on freshwater lake sediment (Fiskal et al., 2019a,b), and marine sediment (Lichtschlag et al., 2011, 2013; Grünke et al., 2012; Felden et al., 2013). These data were filtered to include only depths below 0 mm, and measurements of 0 μM O_2_ were modified to 1 nM to permit the calculation of ΔG without an infinite value. In the case of data from the Nordic Margin (Lichtschlag et al., 2011), the O_2_ concentration never reached a stable zero, hovering around 0–2 μM even below several centimetres of sediment depth. I interpreted this as a technical error, and set the concentration to 1 nM from the depth where the concentration was consistently at its minimum (2.7 cm depth). For the Amon Mud Volcano dataset, the pH data missing from the deepest measurement in one dataset was set to 7.98, to assume that the relatively stable downcore trend would have continued had measurements been made.

Code for the model is available at https://github.com/ianpgm/EMCC_models.

## 3 Results and discussion

### 3.1 A general description of electromicrobiological concentration cells (EMCC)

**Figure 1:**
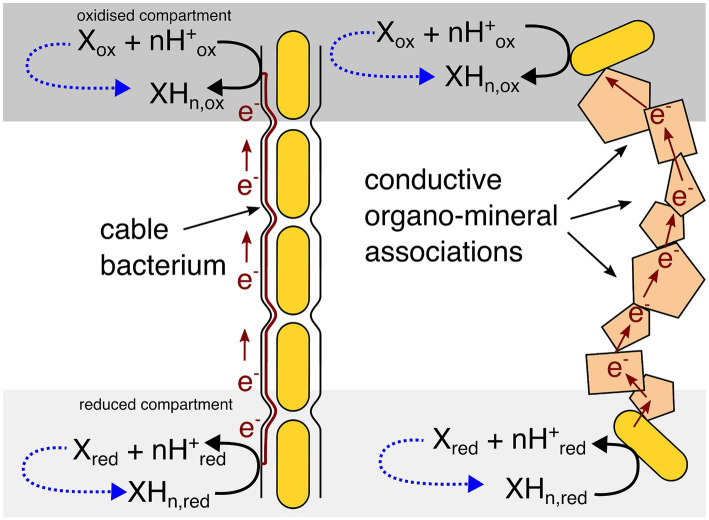
Generic overview of the electromicrobiological concentration cell model. The oxidized and reduced compartments may be a consequence of a concentration gradient (as the examples in this paper show) or a physical conductive barrier preventing diffusion from one compartment to the other. Dotted blue lines show that some other abiotic or biotic process will need to maintain the concentration difference for an EMCC to remain energetically favourable over time.

The EMCC concept, outlined in Figure 1, can seem counter intuitive at first: a reaction where substrates and products are the same is usually understood to result in no net changes in any chemical concentrations and therefore no possibility for an organism to conserve energy. However, the key here is that the substrates and products are in fact not the “same” as they exist in areas with different substrate and product concentrations. The reaction is driven forward by coupling the two reactions together through electrical conductivity, allowing the concentration difference to drive the reaction and permit energy conservation. This concentration difference would have to be maintained by some other biotic or abiotic process to keep the EMCC running, as the EMCC will diminish this concentration difference over time. This is just like any other chemotrophic microorganism—substrates are used up over time, leading to the cessation of metabolism if substrates are not replenished.

**Figure 2:**
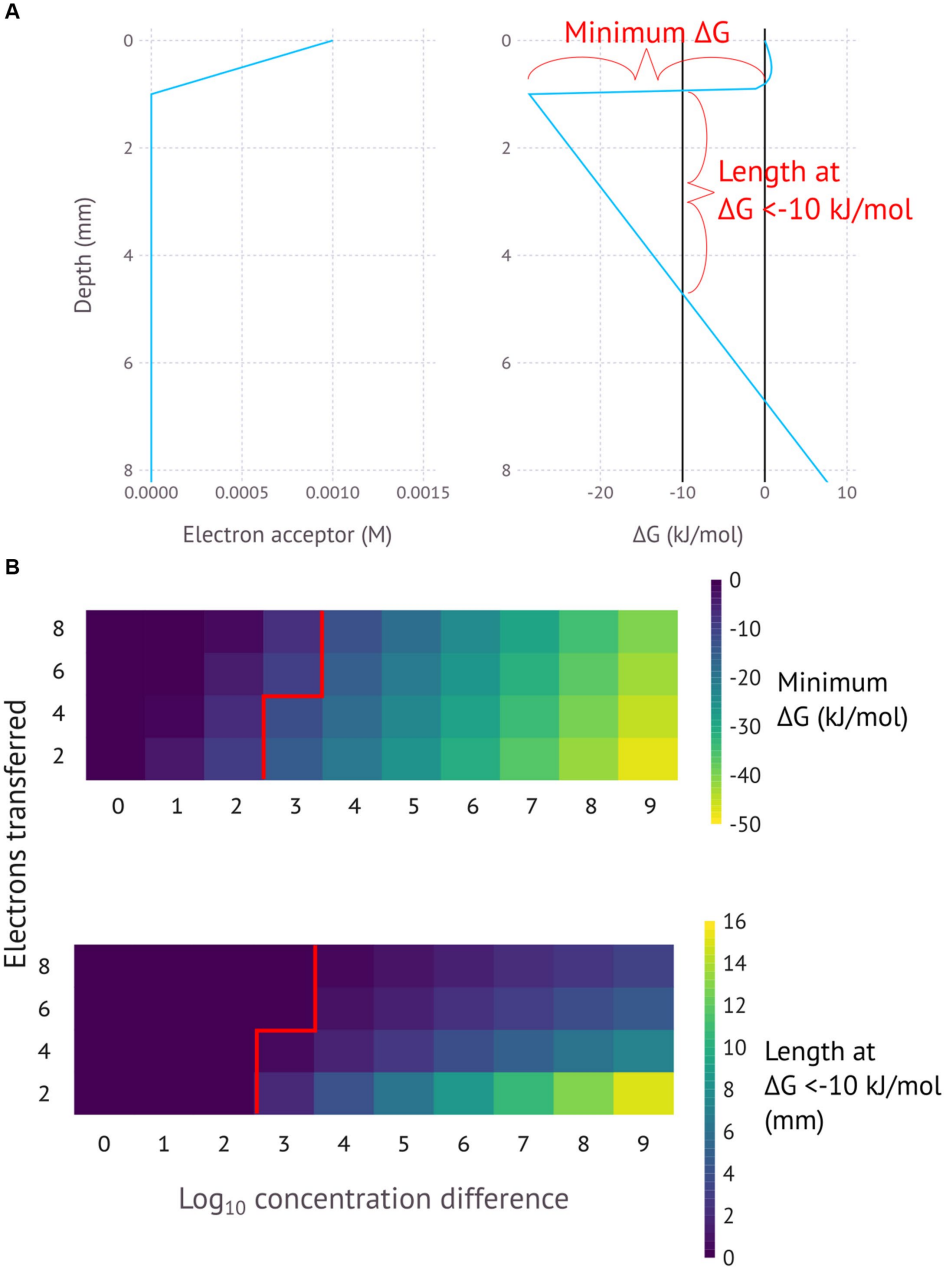
**(A)** General model for an electromicrobiological concentration cell for a 4-electron reaction with 6 orders of magnitude concentration difference, with a minimum. **(B)** General models for a range of different electron numbers and concentration differences, with the minimum Gibbs free energy and distance at <−10 kJ/mol (the assumed minimum necessary for ATP production) shown for reactions with different numbers of electrons transferred and different concentration ranges. pH is assumed to be constant.

Figure 2 presents a generalised model for this process, showing how different variables can affect the ∆G. The assumption is that the electron acceptor varies in concentration over a distance of 1 millimetre, with the lowest concentration being 1 nM and the highest concentration being a number of orders of magnitude above 1 nM. This electron acceptor is reduced by a certain number of electrons to make an electron donor. The calculated Gibbs free energy is for the difference between the concentration at 0 mm and the concentration at the indicated depth. The energy conservation itself does not necessarily occur at the indicated depth, but the indicated ∆G shows the total free energy available for an EMCC extending over that depth. The exact energy yield at each depth would depend on whether the energy was conserved in cells carrying out the oxidation reaction or the reduction reaction.

Figure 2 provides several key insights into the EMCC concept. Firstly, order-of-magnitude changes in concentration differences lead to linear changes in Gibbs free energy. Secondly, the number of electrons transferred change the nFED term in the ∆G calculation and thus alter the slope in the linear part of the calculation. Fewer transferred electrons mean less energy loss along the conductor, resulting in a longer distance that a favourable ∆G can be maintained. This difference in energy loss rate reflects the fact that higher currents result in higher potential loss according to Ohm’s law (V = IR)—assuming a constant substrate turnover rate and resistance, redox reactions transferring more electrons will have a proportionally higher current that results in a higher loss in potential. Put together this means that the most energetically favourable EMCCs involve high concentration differences and low numbers of electrons transferred.

**Figure 3:**
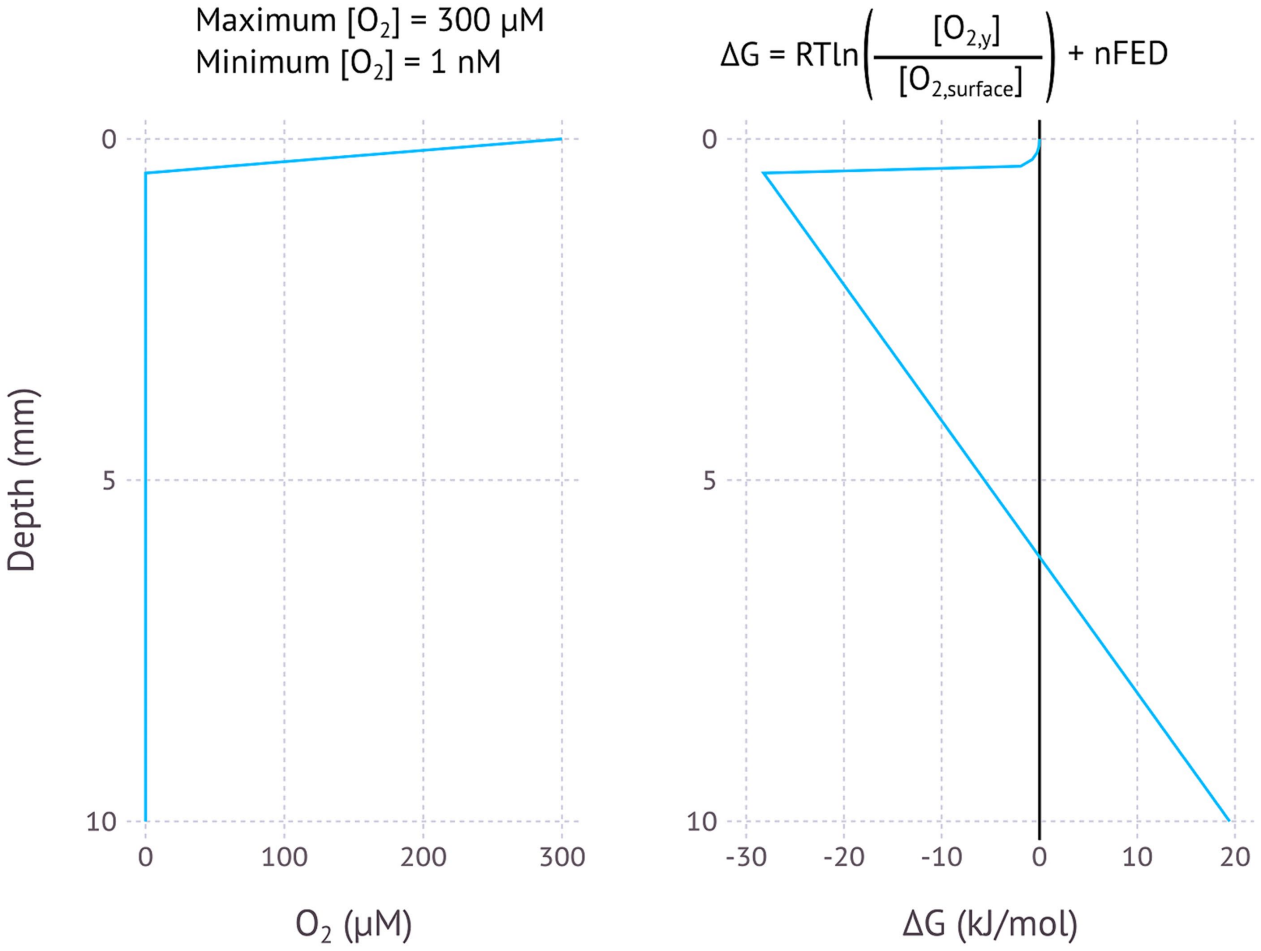
Modelled EMCC based on an O_2_ concentration gradient, assuming an oxygen penetration depth of 0.5 mm, a maximum O_2_ concentration of 300 μM and a minimum concentration of 1 nM. pH is assumed to be constant.

**Figure 4:**
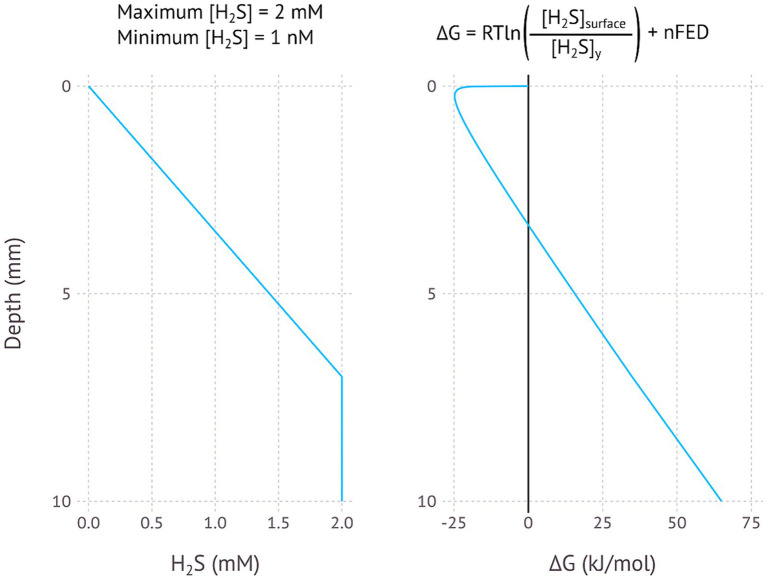
Modelled EMCC based on a H_2_S concentration gradient, assuming most sulfide is removed in the upper 7 millimetres, a maximum H_2_S concentration of 2 mM and a minimum concentration of 1 nM. pH is assumed to be constant.

**Figure 5:**
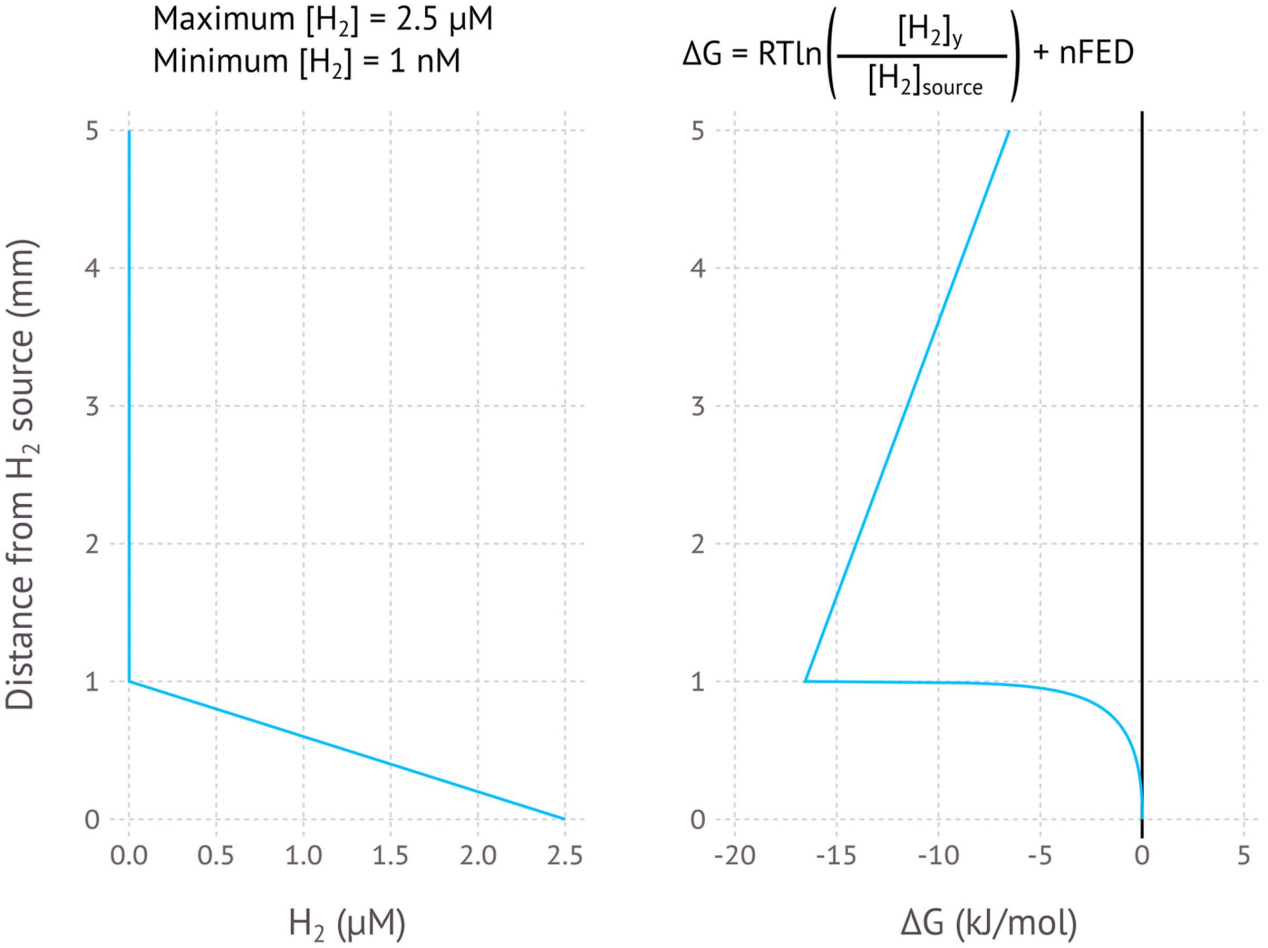
Modelled EMCC based on a H_2_ gradient, assuming H_2_ is produced and concentration diminishes within 1 mm of the production surface. Maximum H_2_ concentration is 2.5 μM, minimum H_2_ concentration is 1 nM.

The model for EMCCs in Figures 1, 2 is best understood when applied to certain specific scenarios, some where the model may help to explain certain difficult to understand observations. Figures 3–5 illustrate models for EMCCs based on O_2_, H_2_S and H_2_ gradients, with the concentration of all other chemical species assumed as constant. These gradients transition from maximum concentration to minimum concentration linearly across a given penetration depth. Penetration depths and maximum substrate concentrations were chosen based on observed literature values (Jørgensen et al., 1979; Nielsen et al., 2015). Supplementary Figures show ∆G calculated from real-world microprofiles for O_2_, S^2−^, and pH – this is a small selection of possible models based on publicly available data, and should not be interpreted as representative of all real-world possibilities. No suitable publicly available H_2_/pH microprofile datasets were found. Choosing a minimum substrate concentration presented an interesting challenge – setting this to 0 μM would make the ∆G impossible to calculate, as the logarithm of zero is infinite. We therefore need to choose an arbitrarily low number for our minimum substrate concentration, but how low this arbitrarily low number is will have a big impact on the resulting ∆G – each order of magnitude change in the minimum concentration could lower the ∆G by up to −5.6 kJ/mol. For each model I have chosen to set the minimum concentration to 1 nM, which is below the 2–20 nM detection limit for the current most sensitive microscale detection methods for O_2_ (Revsbech et al., 2009), H_2_S (e.g., https://unisense.com/products/h2s-microsensor/), and H_2_ (Nielsen et al., 2015) and therefore our best understanding of “zero” at present.

One interesting aspect of EMCCs is the large impact of pH on the resulting ∆G. Protons are produced where oxidation takes place, and consumed where reduction takes place. Multiple protons lead to exponents on the proton concentration in the ∆G calculation, leading to exponential impacts on the overall calculation: an O_2_/H_2_O EMCC involves the transfer of 4 electrons and thus 4 protons, with [H^+^]^4^ then appearing in the numerator and denominator of the reaction quotient. The H_2_S/SO_4_^2−^ EMCC involves 8 protons and the H_2_/H^+^ EMCC involves 2 protons. This means that the pH concentration profile of an environment has a large impact on whether an EMCC is possible. A lower pH at the reducing environment than the oxidizing environment will inhibit an EMCC, while a higher pH in the reducing environment will make an EMCC more likely to be exergonic. Figure 2 and the other theoretical models in this study assume a constant pH, but the models based on real-world data (Supplementary Figures) take pH differences into account and show that in many cases the reaction could still be thermodynamically favourable even when the reducing end of the EMCC has a lower pH. As an EMCC runs, more protons are produced at the reducing end and consumed at the oxidizing end, making the pH profile less favourable for an EMCC as time goes on, with the rate of this degradation in ∆G a function of how well buffered the system is. In a poorly buffered system, the pH difference will render the reaction unfavourable before the other system components due to the higher exponents.

The calculated changes in Gibbs free energy closest to the zero position are a function of whether the chemical species at the zero position is consumed (O_2_ and H_2_) or produced (H_2_S), with consumption resulting in a concave curve and production in a convex curve. The linear part of the curve starts where the concentration becomes constant – here the energy change is a function of the distance and the number of electrons transferred, with more electrons resulting in a steeper slope (Figures 2–5).

### 3.2 Oxygen reduction coupled to water splitting


$$2H_{2}O_{red}+4H_{ox}^{+}+O_{2,ox}\to 2H_{2}O_{ox}+4H_{red}^{+}+O_{2,red}$$

$$\Delta G=RT\ln\left(\frac{{\left[H_{red}^{+}\right]}^{4}\left[O_{2,red}\right]}{{\left[H_{ox}^{+}\right]}^{4}\left[O_{2,ox}\right]}\right)+nFED$$

The oxic/anoxic interface in aquatic sediments is a possible environment where EMCCs may occur, as shown by the theoretical model used here (Figure 3) and the models based on O_2_ and pH microprofiles from freshwater and marine environments (Supplementary Figures SF1–F6). Oxygen from the water column is consumed by aerobic microorganisms, often in the uppermost millimetre of sediments with ample electron donor. This creates a steep O_2_ gradient in the sediment, meaning that a conductive structure could connect O_2_-reducing and H_2_O-oxidizing processes on either end of this gradient without losing too much potential.

The steepness of the O_2_ gradient appears to be critical for whether or not an EMCC will be possible – O_2_ penetration depths of about 1–2 mm as found in Lake Baldegg (Supplementary Figure SF1), Lake Greifen (Supplementary Figure SF2), the shallow part of Lake Lucerne (Supplementary Figure SF3), Lake Zug (Supplementary Figure SF4), and Amon Mud Volcano (Supplementary Figure SF5) result in ∆G values below −10 kJ/mol, while O_2_ penetration depths of about 1 cm or more found in the two deep cores from Lake Lucerne (SF3) and the Nordic Margin (SF6) result in positive ΔG values. This is because the potential difference lost over the longer O_2_ penetration depth is greater than the potential difference created by the concentration difference. The fact that steeper gradients are more likely to produce EMCCs introduces an interesting paradox – O_2_ is depleted rapidly by aerobic microorganisms oxidizing organic matter, and more organic matter (such as in the eutrophic lakes Baldegg, Greifen, and Zug) results in a steeper decline in O_2_ concentration than environments with less organic matter, like Lake Lucerne. However, higher organic matter concentrations will also mean that an EMCC microorganism is more likely to be outcompeted by an aerobic chemoorganotroph in the oxic sediment layer. The EMCC organism could only become established if there was some factor preventing direct competition for oxygen, such as physically extending further into the oxic zone above the sediment surface. Similar processes have been observed in cable bacteria emerging from the sediment under conditions of oxygen limitation (Burdorf et al., 2018).

Cable bacteria form conductive structures from the oxic layer to the anoxic layer, allowing an exergonic production of O_2_ from water in the uppermost part of the anoxic layer. Such O_2_ production may explain the observation of so-called “flocking” bacteria, microorganisms capable of aerobic respiration that flock around cable bacteria in the anoxic zone of a slide designed to reproduce O_2_ gradients as in sediment (Bjerg et al., 2023; Lustermans et al., 2023). These flocking bacteria would consume O_2_ produced by the cable bacteria, keeping the O_2_ concentration in the anoxic sediment low and allowing the O_2_-producing reaction to continue to proceed. In a way this would extend the influence of O_2_ several millimetres below the oxic/anoxic interface, even allowing O_2_-dependent reactions such as aerobic methane and ammonium oxidation to proceed. There is currently no direct evidence for such cryptic O_2_ production in sediment, only some evidence of O_2_ production by cable bacteria filament sheaths in an *in vitro* concentration cell (Digel et al., 2023), but the model presented here shows that such a process is thermodynamically possible.

These thoughts about O_2_ production in cable bacteria come amid an increasing wave of interest in trace amounts of O_2_ produced in ostensibly anoxic environments and making aerobic respiration possible (Berg et al., 2022; Kraft et al., 2022; Ruff et al., 2023). While EMCCs cannot explain many of the observations made until now, especially in the water column, the potential for such concentration-driven production of O_2_ ought to be considered a possible explanation for some otherwise difficult-to-explain aerobic metabolism.

### 3.3 Sulfate reduction coupled to sulfide oxidation


$$\begin{array}{l} 4H_{2}O_{red}+H_{2}S_{red}+8H_{ox}^{+}+{\mathrm{SO}}_{4}^{2-}{\;}_{ox}\to 4H_{2}O_{ox}\\ +H_{2}S_{ox}+8H_{red}^{+}+{\mathrm{SO}}_{4}^{2-}{\;}_{red} \end{array}$$

$$\Delta G=RT\ln\left(\frac{\left[H_{2}S_{ox}\right]\;{\left[H_{red}^{+}\right]}^{8}\left[{\mathrm{SO}}_{4}^{2-}{\;}_{red}\right]}{\left[H_{2}S_{red}\right]\;{\left[H_{ox}^{+}\right]}^{8}\left[{\mathrm{SO}}_{4}^{2-}{\;}_{ox}\right]}\right)+nFED$$

Sulfate reduction is a strong candidate for EMCCs, as the dissimilatory sulfite reduction (DSR) pathway is already known to be able to run in reverse, oxidizing sulfide or reducing sulfate. It used to be thought that microorganisms using the oxidative DSR pathway were phylogenetically distinct from sulfate-reducing organisms (Müller et al., 2015), but sulfide-oxidizing organisms with a *dsrAB* genes that belong to sulfate-reducing clades have since been found. These include cable bacteria (Kjeldsen et al., 2019) and *Desulfurivibrio alkaliphilus* (Thorup et al., 2017). Similarly, many sulfate-reducing bacteria have been shown to oxidize sulfide while reducing O_2_, albeit without observed growth (Dannenberg et al., 1992). While a single organism capable of energy conservation using either sulfate reduction and sulfide oxidation has not yet been observed, it has not been ruled out yet either, especially not using an electrode as electron donor or acceptor rather than a chemical substrate. One could imagine a situation where an organism capable of both sulfide oxidation and sulfate reduction could couple these two processes together electrically from an environment with high sulfide concentration to low sulfide concentration (Figure 4). An examination of sulfide and pH gradients in nature shows that in at least one case such a sulfide-driven EMCC would be exergonic (Supplementary Figure SF7).

One interesting consequence of this model would be that abiotic consumption of sulfide could enable an EMCC to use alternative electron donors indirectly. For example, cable bacteria are not known to reduce metal oxides or organic matter, and nor do they have any clear indications in their genome that such electron acceptors could be used (Kjeldsen et al., 2019). However, metal oxide minerals (Peiffer et al., 1992) and organic matter (Yu et al., 2015) can abiotically oxidize sulfide, creating a low sulfide zone around their surfaces. A cable bacteria EMCC could drive its metabolism using this area around a mineral with a lower sulfide concentration against a relatively constant sulfate concentration background, thus indirectly accessing alternative electron acceptors with abiotic sulfide/sulfate cycling as an intermediate. Cable bacteria activity results in iron oxide near the sediment surface (Seitaj et al., 2015), could these iron oxides support cable bacteria survival during annual bottom water hypoxia?

A laboratory analogue to such sulfur-mediated use of alternative electron acceptors may already have been observed. There have been several observations of cable bacteria being attracted to and potentially growing on graphite anodes in the place of other electron acceptors (Reimers et al., 2017; Li et al., 2021; Bonné et al., 2024). Could these graphite electrodes be abiotically oxidizing sulfide released from the sediment, creating a low-sulfide zone around the electrode that can enable the coupling of sulfate reduction close to the electrode to the oxidation of sulfide distant from the electrode? Abiotic sulfide oxidation at graphite electrodes has been observed before (Ateya and Al-Kharafi, 2002). The observed increase in current compared to cable-free controls could be explained by the cable bacteria increasing the effective surface area of the electrode. This explanation would avoid the need for any direct-interspecies electron transfer to the electrodes by cable bacteria.

### 3.4 Proton reduction coupled to hydrogen consumption


$$H_{2,red}+2H_{ox}^{+}\to H_{2,ox}+2H_{red}^{+}$$

$$\Delta G=RT\ln\left(\frac{\left[H_{2,ox}\right]{\left[H_{red}^{+}\right]}^{2}}{\left[H_{2,red}\right]{\left[H_{ox}^{+}\right]}^{2}}\right)+nFED$$

Hydrogen metabolism is widespread amongst prokaryotes, with both H_2_ oxidation and H_2_ production by H^+^ reduction coupled to many other redox reductive processes. Several types of hydrogenases exist to mediate these reactions, and [NiFe]-hydrogenases are known to catalyse both the oxidation of H_2_ and the reduction of H^+^ (Vignais and Billoud, 2007). H_2_ as an electron donor for microorganisms can be produced geologically (Nealson et al., 2005). One could imagine a subsurface source of H_2_ where the H_2_ diffuses away, creating a high-H_2_ to low-H_2_ gradient, ideal for an electromicrobiological concentration cell driven by the H_2_ concentration difference, assuming that pH remains relatively constant (Figure 5).

The idea of an H_2_ EMCC is particularly interesting in the context of the earliest forms of metabolism in the early Earth or other planets. One challenge for the first life forms was the absence of electron acceptors—the early Earth atmosphere was very reduced (Shaw, 2008), and the first life has often been thought to use nitrogen oxides (Mancinelli and McKay, 1988) or carbon dioxide (Walker, 1985). But a H_2_-based concentration cell gives us another possible electron acceptor for early life: H^+^, coupled to the oxidation of H_2_. In this way early life would not have had to find a way to couple energy conservation to CO_2_ fixation all at once (as in the complex methanogenesis pathway), but could have had an alternative energy source from the beginning. One key advantage for early life would be that a single reversible proto-hydrogenase could function both to oxidize the electron donor and reduce the electron acceptor, limiting the complexity for a minimal metabolism to work. Extracellular H_2_ oxidation produces a higher H^+^ concentration outside the cell, generating a proton motive force that could have driven the first metabolism with a single catalyst. This is not the first suggestion that concentration gradients could have helped drive metabolism in the earliest life (e.g., Ooka et al., 2019), but it is striking how simple the earliest life form could be if concentration cells are taken into account.

## 4 Perspectives

While it seems clear that electromicrobiological concentration cells are theoretically possible, and that there are some experimental observations which may be explained by the model described in this paper, experimental tests of this theory will be the next logical step.

Identifying environments where EMCCs may be found can be done based on fine-scale chemical characterisation of heterogeneous aquatic environments. Competition with other members of the microbial community is an important factor in determining whether an EMCC will be established – if other electron donors or acceptors are present in the area where substrates are consumed, other microorganisms may outcompete EMCC microbes. For example, a conventional aerobic organotroph will likely outcompete an O_2_-reducing EMCC at an oxic/anoxic interface with plenty of organic matter available as an electron donor. Or a sulfide-oxidizing EMCC will be outcompeted by a conventional nitrate-reducing sulfide-oxidizer if nitrate is present. The best place to find EMCCs are therefore environments with steep changes in substrate concentration but without too many alternative electron donors or acceptors to use these substrates. Such environments are rare – steep gradients are typically a result of high metabolic rates driven by non-EMCC metabolic processes that will typically outcompete the EMCC organisms, but there are certain situations that have been covered in this paper that would circumvent this problem: EMCC structures that extend beyond the edge of the gradients to an area with less competition, for example, or gradients driven by physical or chemical processes rather than metabolism. EMCCs based on chemical gradients produced by other microbes will likely have a minimal impact on overall geochemical cycles, as the processes producing these gradients will be operating at higher rates. While the observation of chemical gradients at the micrometre to millimetre scale has improved vastly over recent decades with microsensors, optodes, and other technologies, there are still many environments, particularly at geochemical transition zones in the deep subsurface, that have not been well characterised on the micro-scale for logistical reasons.

Once potential EMCC environments are identified, the next step would be to identify possible microorganisms involved. While cultivation and isolation has been the most effective method of understanding microbial physiology for over a 100 years, it is obvious why no EMCC-organism has ever been isolated: most cultivation systems are homogeneous and mixed, and the relatively few microbiologists who have carried out gradient-based enrichment and isolation (for example for the cultivation of colourless sulfur bacteria) have used opposing gradients of different electron donors and acceptors, rather than a gradient of the same chemical species from its oxidized to its reduced form. Such EMCC gradient systems would be even more challenging to operate than traditional gradient systems, so either a great deal of effort or some technical innovation (probably both) would be necessary for this to work. Perhaps bioelectrochemical systems similar to the sediment microbial fuel cells described above for cable bacteria could be the way forward for enrichment and cultivation.

If enrichment and isolation of EMCC microorganisms is difficult, could their direct observation in the environment or laboratory microcosms be the way forward? Electrochemical and optical sensing is improving constantly and may provide new opportunities for measuring gradients at the millimetre to centimetre scale. The electric potential microelectrode (Damgaard et al., 2014) has proven vital for cable bacteria research, and EMCCs ought to produce an electric field just as conventional cable bacteria metabolism does, so the electric potential microelectrode could play a similar role. Other forms of sensing, such as stable-isotope based methods and living biosensors, could also be important. With observations of EMCCs, more accurate models than those presented here will become possible. For example, the energy loss along the conductor, critical for determining whether en EMCC is exergonic or not, is a function of the current through the conductor – if this current can be estimated, then more accurate predictions could be made. EMCCs operating at very low rates could be exergonic for much longer distances than the predictions made in this study.

## 5 Conclusion

Electromicrobiological concentration cells are fascinating theoretical constructs which, if found to exist in the real world, could greatly impact our understanding of microbial element cycling, physiology, and the origin of life on Earth and elsewhere. I hope that other scientists will be inspired by this paper to explore this concept further, both theoretically and experimentally.

## Data Availability

The original contributions presented in the study are included in the article/Supplementary material, further inquiries can be directed to the corresponding author.
