# Supplementary material for: Electromicrobiological concentration cells are an overlooked potential energy conservation mechanism for subsurface microorganisms
Source: Front Microbiol. 2024 Aug 21;15:1407868. doi: 10.3389/fmicb.2024.1407868 (PMC11371792; doi:10.3389/fmicb.2024.1407868)

SF1

Lake Baldegg core LB_1 (66m water depth)

https://doi.pangaea.de/10.1594/PANGAEA.908524

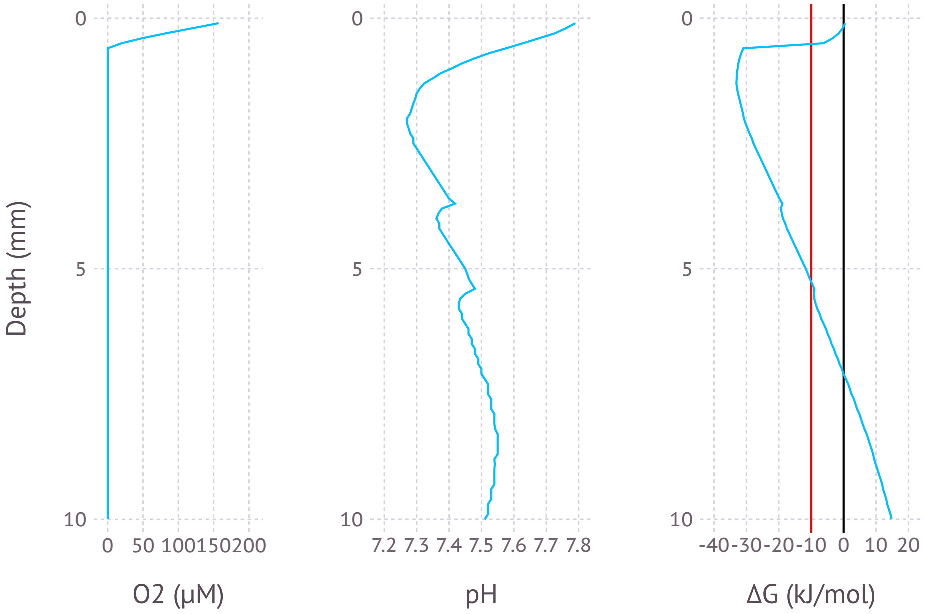

Lake Baldegg core LB_2 (45m water depth)

https://doi.pangaea.de/10.1594/PANGAEA.908526

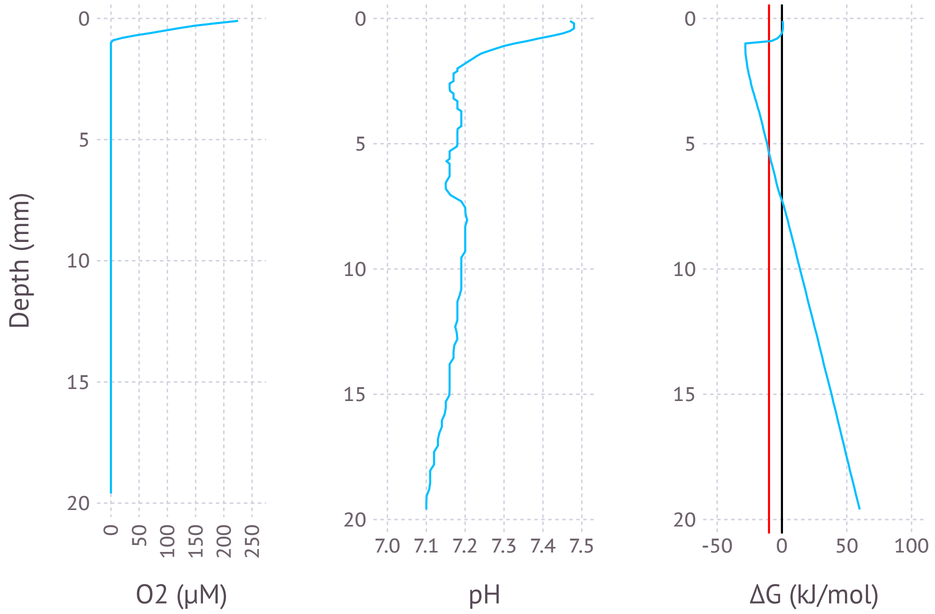

Lake Baldegg core LB_3 (21m water depth)

https://doi.pangaea.de/10.1594/PANGAEA.908527

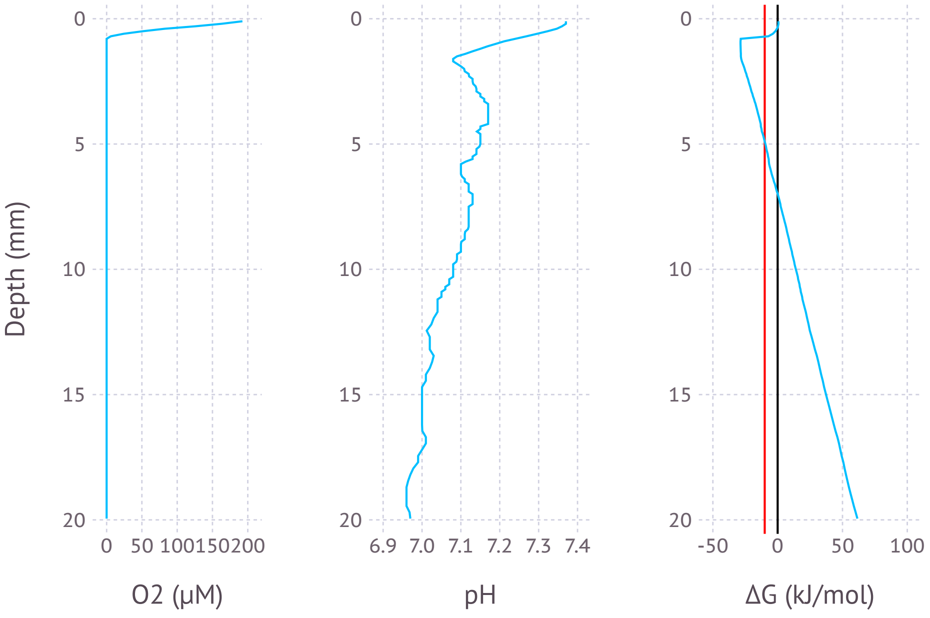

Supplemental figures SF1 - SF7 show calculated EMCC Gibbs free energy yields based on microprofiles for pH and O2 (SF1-6) and S2- (SF7). The black vertical line indicates 0 kJ/mol, the red vertical line indicates -10 kJ/mol. Source data sets and metadata from Pangaea are accessible via the provided DOI links.

SF2

Lake Greifen LG_1 (15m water depth)

https://doi.pangaea.de/10.1594/PANGAEA.908528

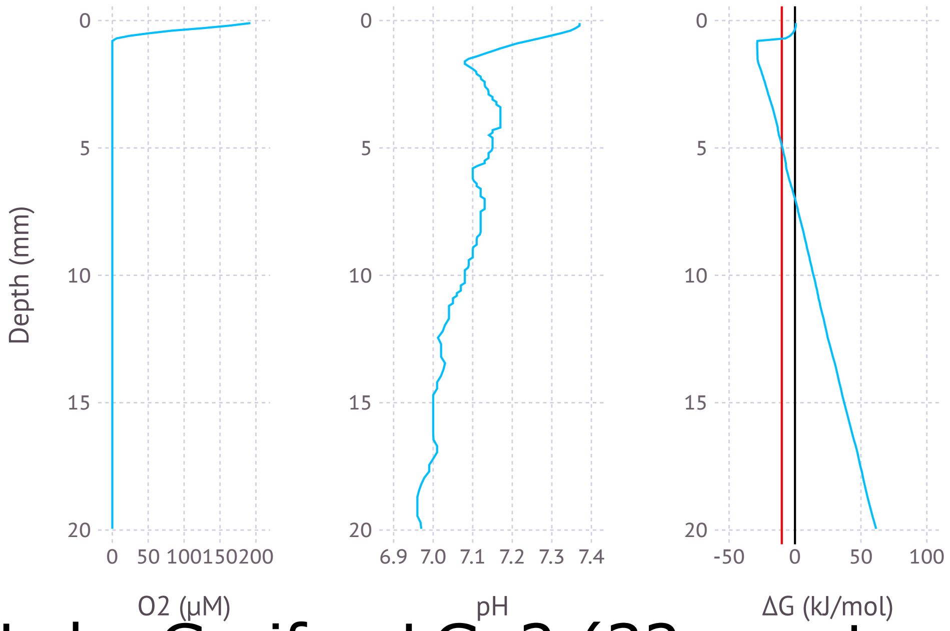

Lake Greifen LG_2 (32m water depth)

https://doi.pangaea.de/10.1594/PANGAEA.908529

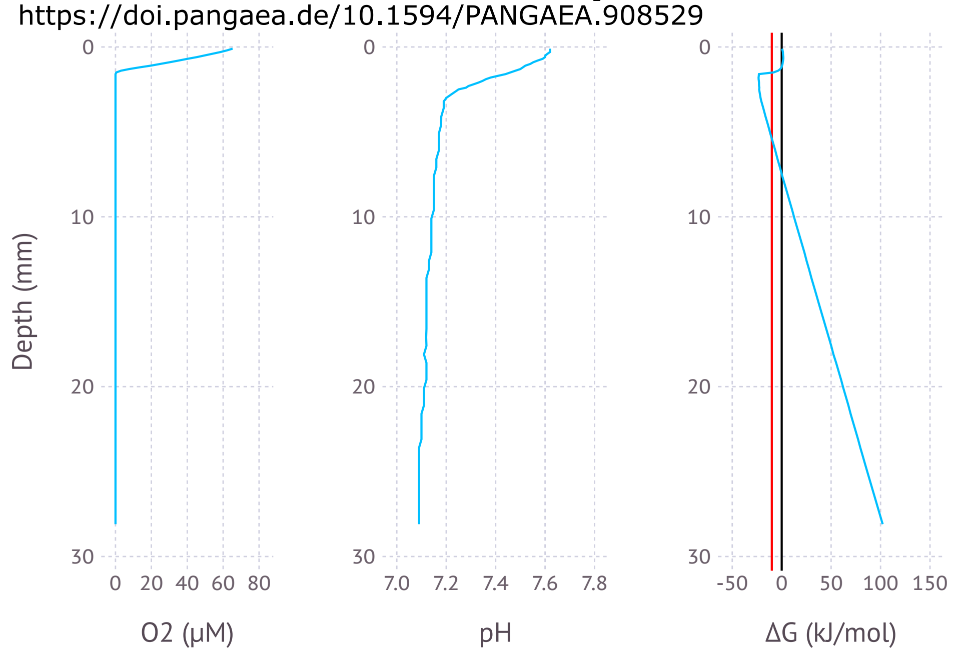

Lake Greifen LG_3 (24m water depth)

https://doi.pangaea.de/10.1594/PANGAEA.908530

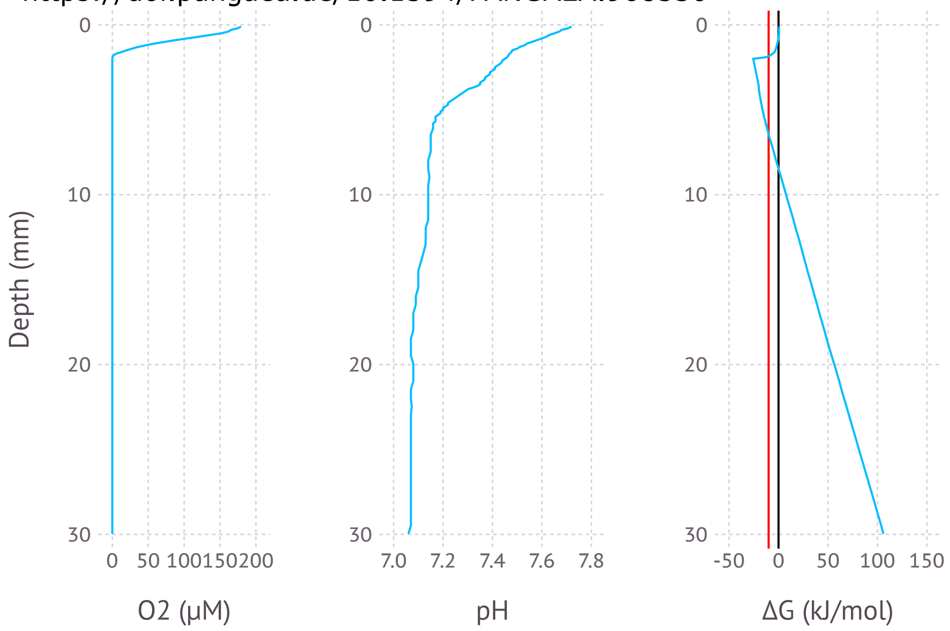

SF3

Lake Lucerne LL_1 (24m water depth)

https://doi.pangaea.de/10.1594/PANGAEA.908531

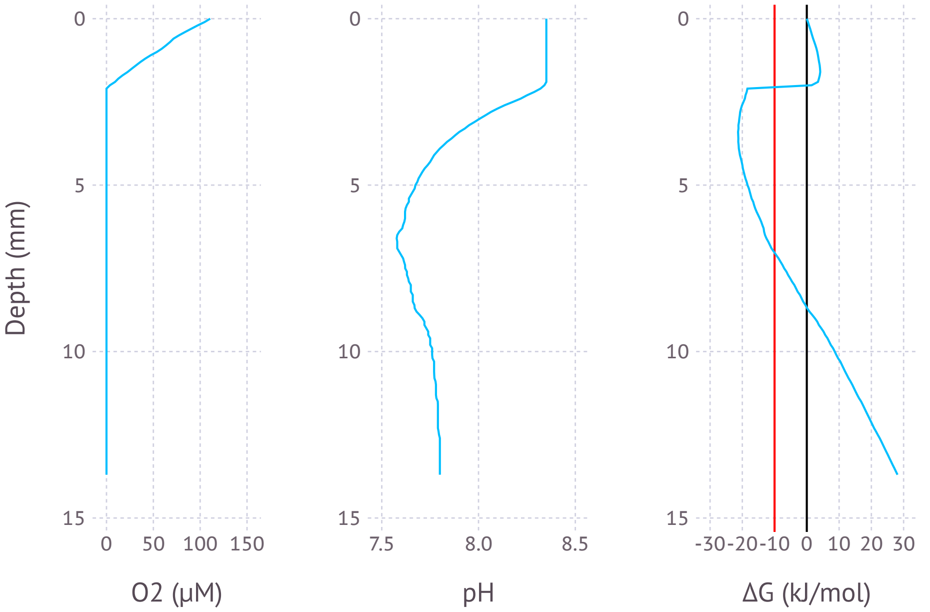

Lake Lucerne LL_2 (93m water depth)

https://doi.pangaea.de/10.1594/PANGAEA.908532

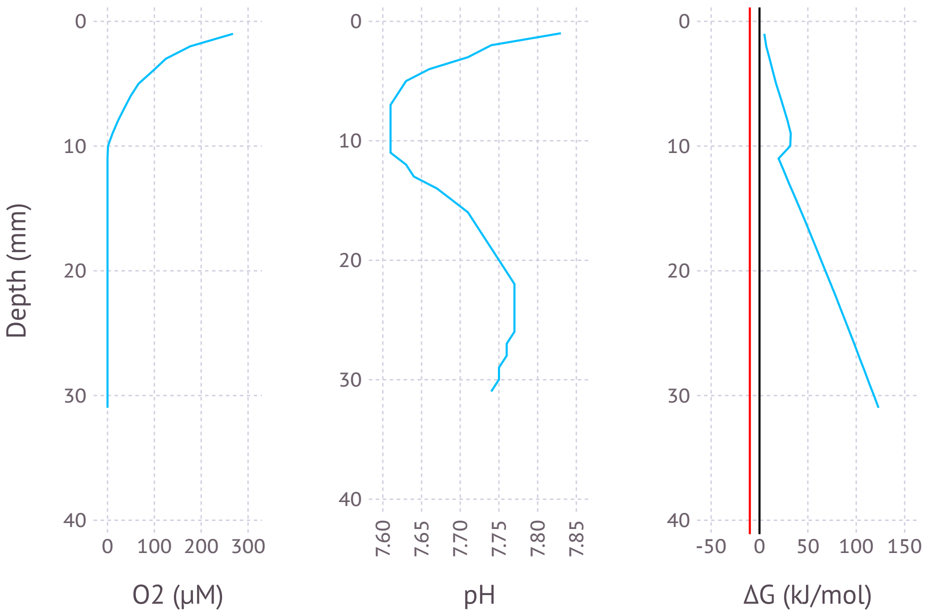

Lake Lucerne LL_3 (45m water depth)

https://doi.pangaea.de/10.1594/PANGAEA.908533

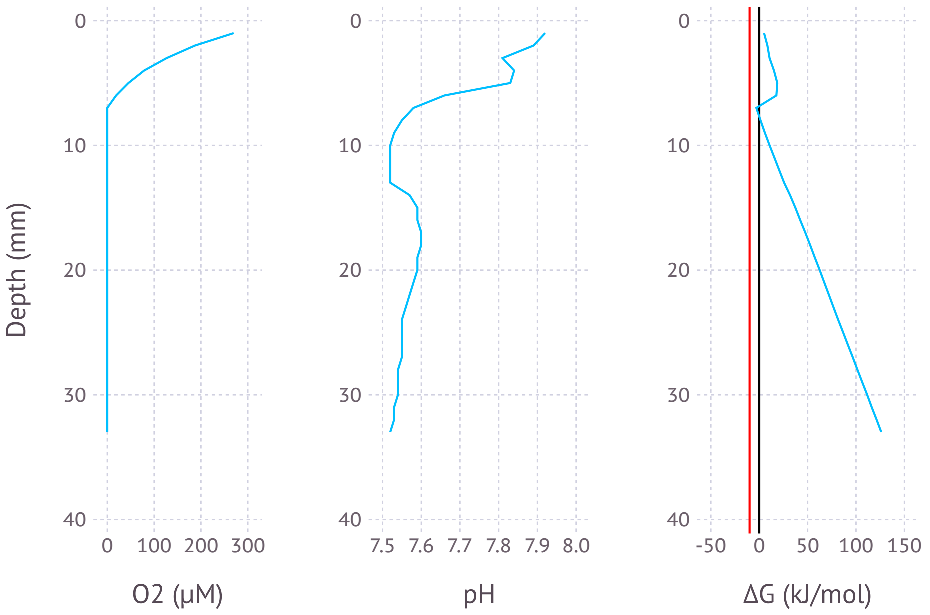

SF4

Lake Zug LZUG_1 (25m water depth)

https://doi.pangaea.de/10.1594/PANGAEA.908537

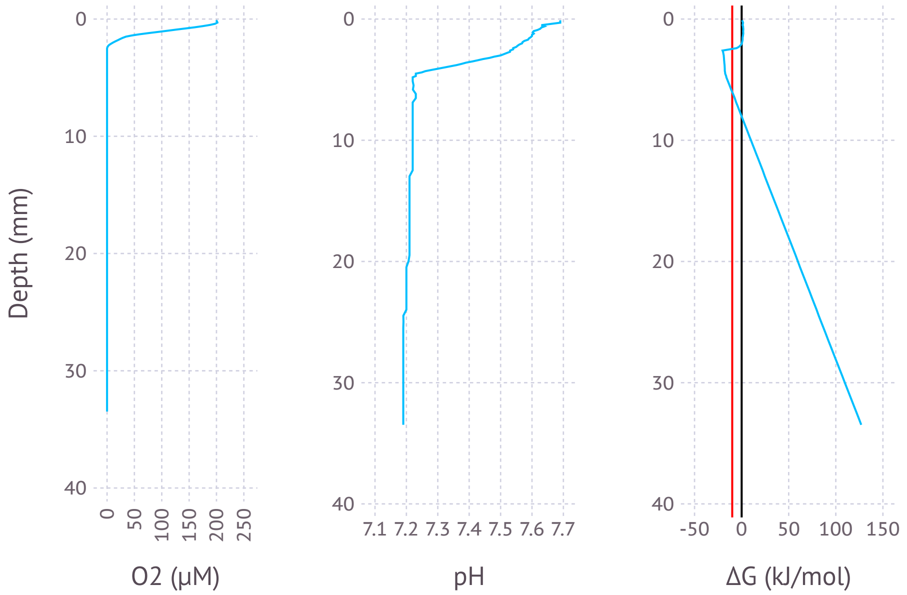

Lake Zug LZUG_2 (35m water depth)

https://doi.pangaea.de/10.1594/PANGAEA.908540

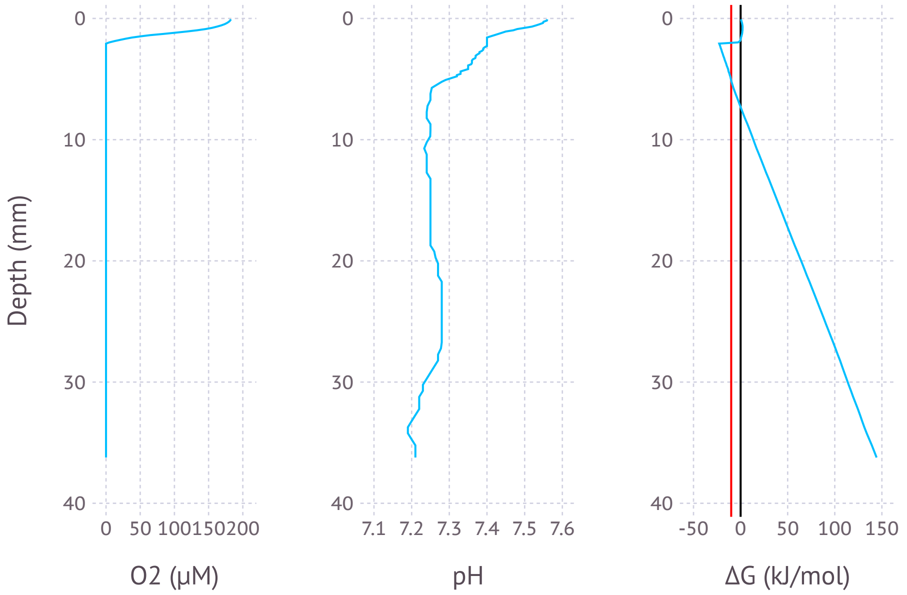

Lake Zug LZUG_3 (50m water depth)

https://doi.pangaea.de/10.1594/PANGAEA.908541

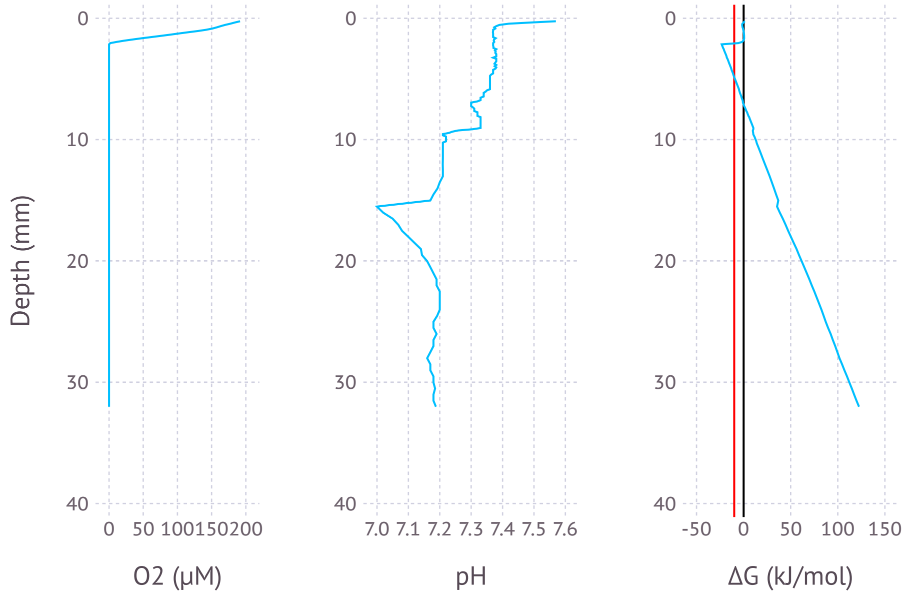

SF5

Amon Mud Volcano M70/2b_825_PROF-1

https://doi.pangaea.de/10.1594/PANGAEA.809982

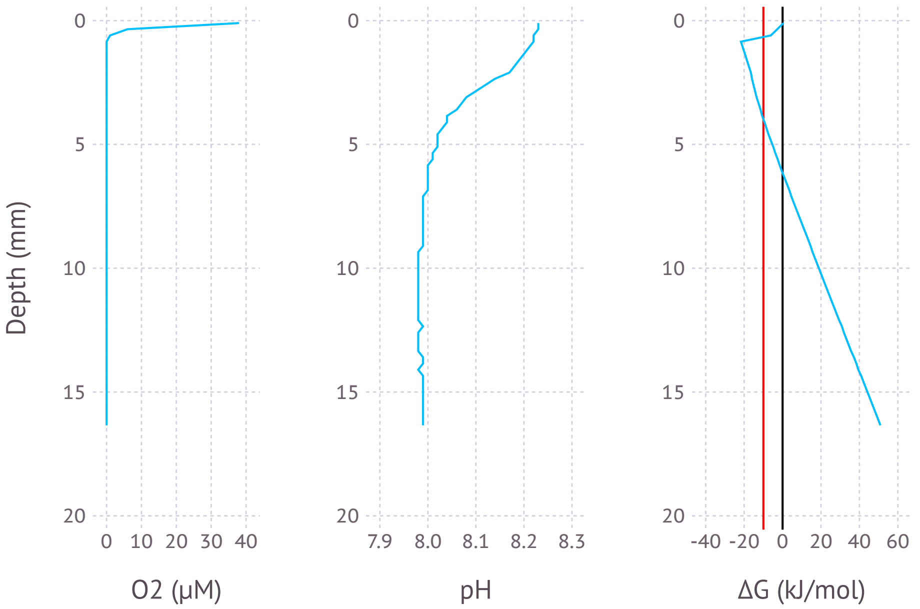

Amon Mud Volcano M70/2b_805_PROF-2

https://doi.pangaea.de/10.1594/PANGAEA.809994

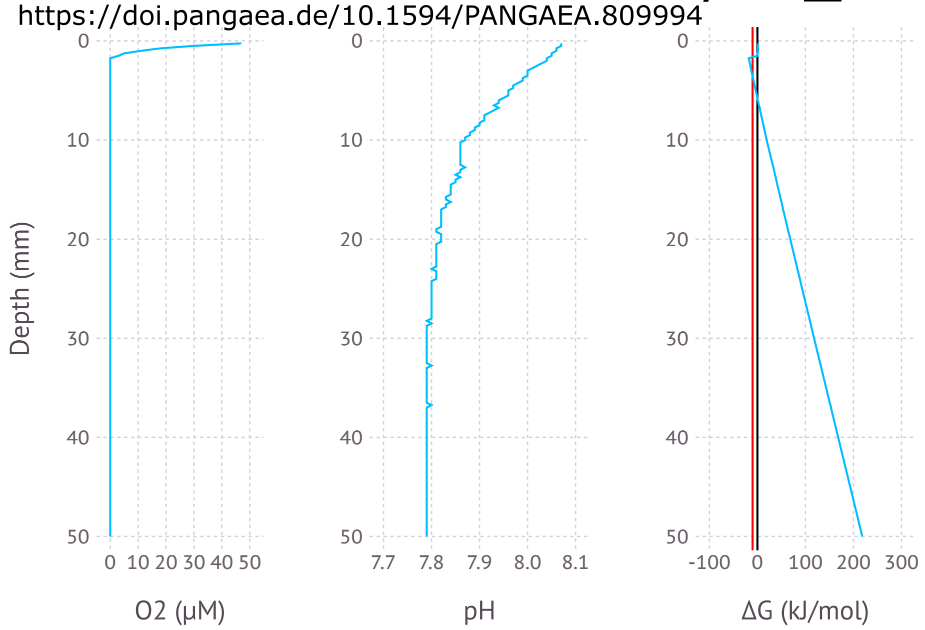

Amon Mud Volcano M70/2b_790_MICP-1

https://doi.pangaea.de/10.1594/PANGAEA.809984

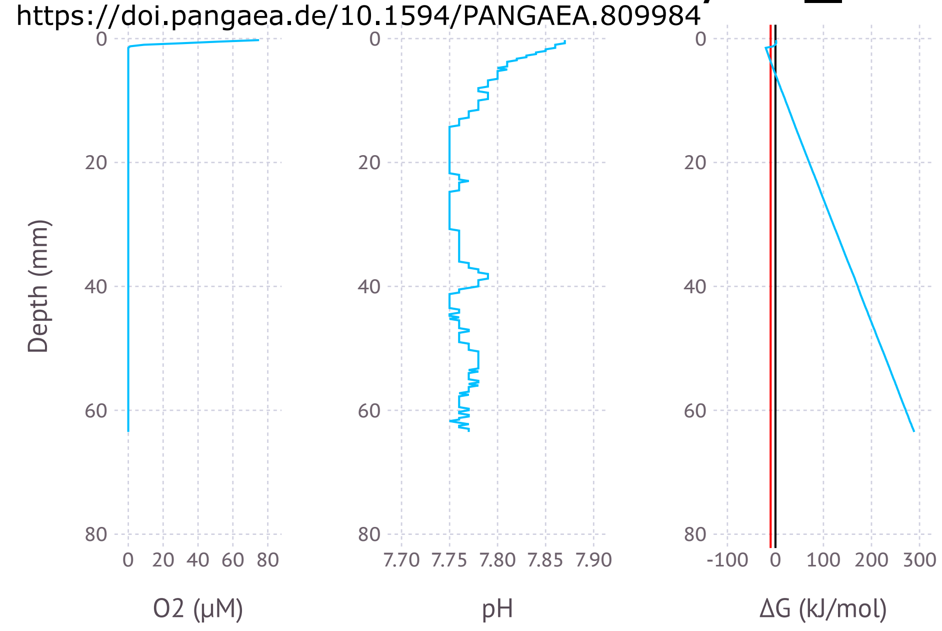

SF6

Nordic Margin VKGD272/MIC-2

https://doi.pangaea.de/10.1594/PANGAEA.772691

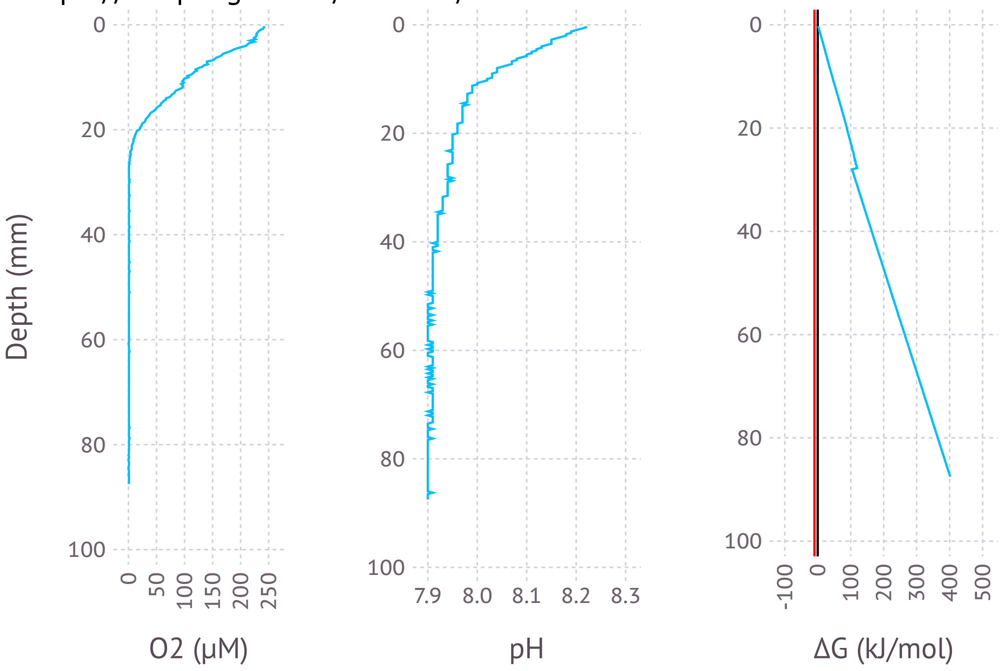

SF7

Amon Mud Volcano M70/2b_825_PROF-1

https://doi.pangaea.de/10.1594/PANGAEA.809982

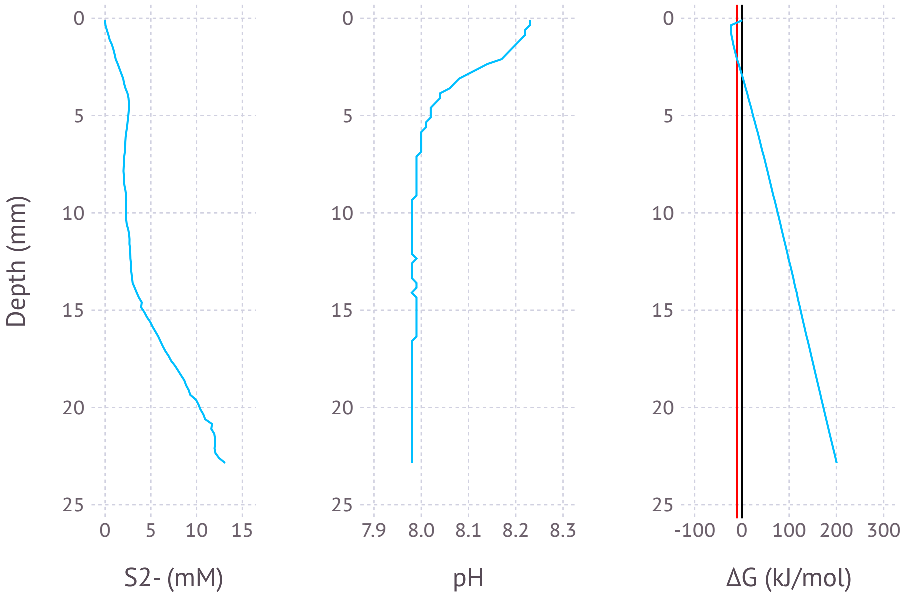

Amon Mud Volcano M70/2b_805_PROF-2

https://doi.pangaea.de/10.1594/PANGAEA.809994

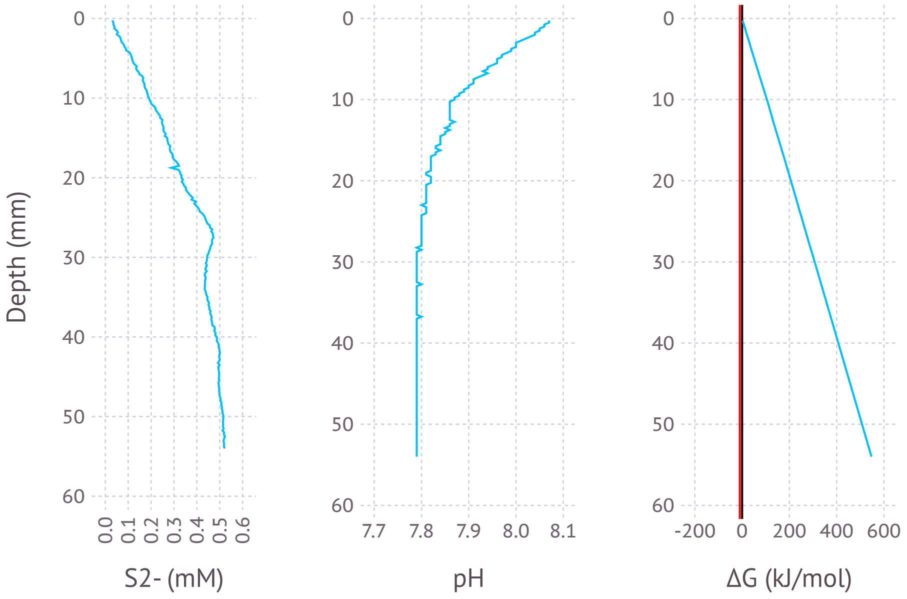

Amon Mud Volcano M70/2b_790_MICP-1

https://doi.pangaea.de/10.1594/PANGAEA.809984

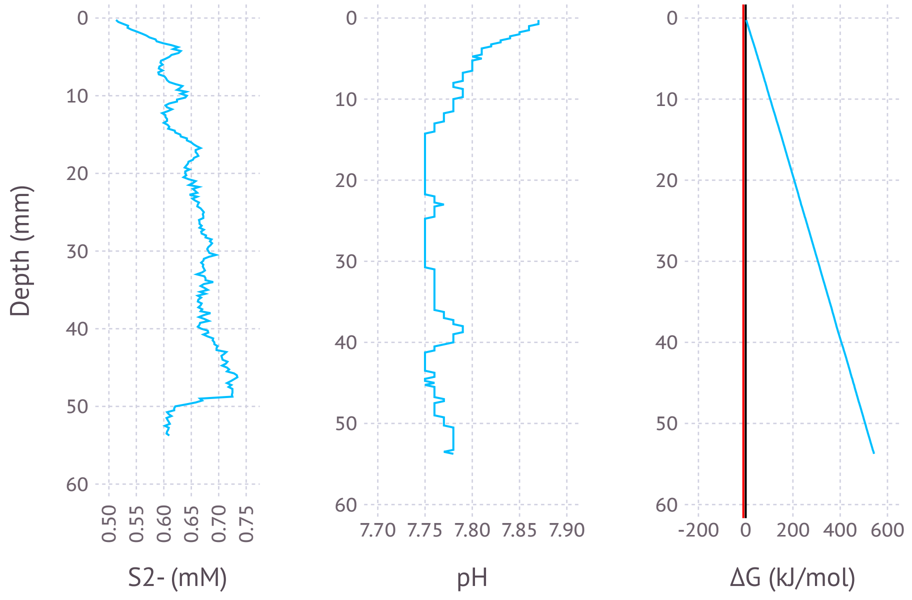

Supplement: Supplementary file 1 [file Data_Sheet_1.PDF]
